# Gaucher Disease: New Expanded Classification Emphasizing Neurological Features

**Published:** 2019

**Authors:** Mohammad Reza ALAEI, Aydin TABRIZI, Narjes JAFARI, Hadi MOZAFARI

**Affiliations:** 1 Pediatric Endocrinology, Faculty of Medicine, Shahid Beheshti University of Medical Sciences, Tehran, Iran; 2 Pediatric Neurology Research Center,Research Institute for Children’s Health, Shahid Beheshti University of Medical Sciences, Tehran, Iran.; 3 Pediatric Biochemistry, Medical school, Kermanshah University of Medical Sciences, Kermanshah, Iran

**Keywords:** Gaucher disease, Neurological manifestations, Phenotypes

## Abstract

Gaucher disease (GD) is a rare inherited metabolic disorder and the most common lysosomal storage disorder, caused by a deficiency in glucocerebrosidase enzyme activity. It has been classified according to the neurological manifestations into three types: type 1, without neuropathic findings, type 2 with acute infantile neuropathic signs and type 3 or chronic neuropathic form. However, report of new variants has led to the expansion of phenotype as a clinical phenotype of GD considered as a continuum of phenotypes. Therefore, it seems that a new classification is needed to cover new forms of the disease.

## Introduction

Lysosomal storage disorders (LSDs) are a different group of nearly sixty inherited metabolic diseases (1) characterized by an accumulation of harmful products in the lysosomes because of malfunction of its specific proteins. As a result of a lysosomal dysfunction, cellular function disrupts and clinical abnormalities appear subsequently. In respect of the intracellular depository material, LSDs can be divided into three major groups including the sphingolipidoses, mucopolysaccharidoses, and glycoproteinoses (2). 

Gaucher disease (GD, OMIM #230800, ORPHA355) is the most common sphingolipidoses (1, 3) with an accumulation of the toxic amounts of certain fatty materials- glucosylceramide- primarily within the lysosomes of macrophages in the diverse tissues, transforming macrophages into storage cells named Gaucher cells throughout the body (4, 5). 

## History

GD was first recognized by a French doctor in 1882 (6). He described a young female patient with a huge splenomegaly without evidence of malignancy rather the presence of largely unusual cells in the involved spleen. After reporting other similar patients, the autonym “Gaucher’s disease” was denoted (7). Neurologic impairment in an infant was first reported in 1927(8). This phenotype eventually became known as the infantile or acute neuronopathic form (9). In the 1960s, the pathogenesis of GD was described: A functional deficiency of β-glucocerebrosidase activity as the primary pathomechanism (10). 

## Classification

**Figure 1:**
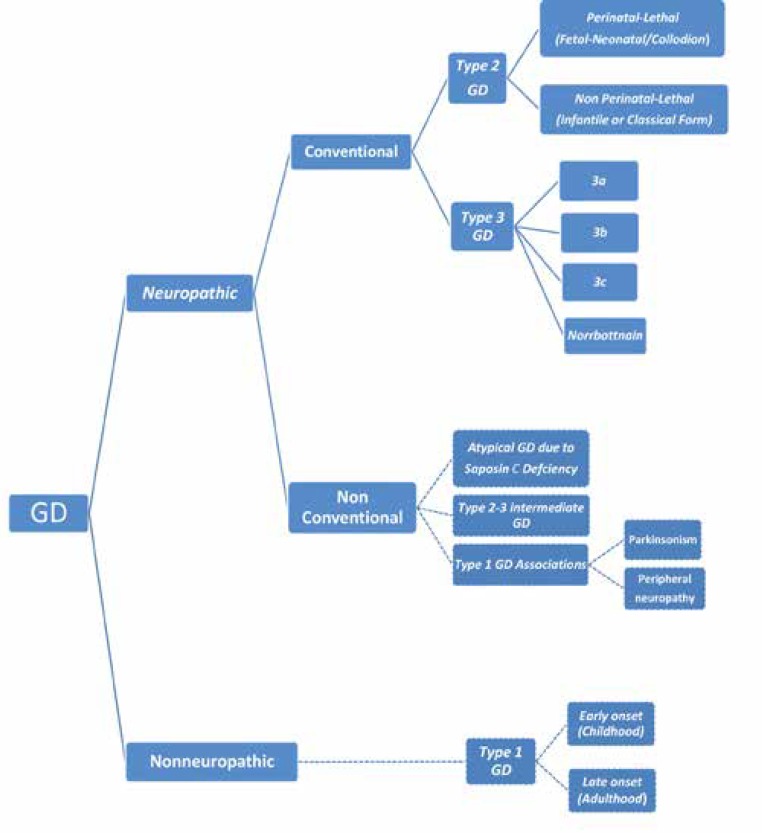
New expanded classification of GD

GD is classically divided into two major clinical subgroups and three main subtypes (Figure 1) according to the absence or presence of neurological involvement with its severity and deterioration, age at determination and progression rate (11-13). 

**1) Non-neuronopathic group**

This group includes Type 1 GD (OMIM #230800; ORPHA: 77259)’ also known as adult and chronic GD (14, 15) which is the most prevalent subtype in the Western countries (12, 16, 17). It is characterized by the visceral presentations without CNS involvement (18) distinguishing from the other two types in neuropathic group (19). 

**2- Neuronopathic group**

Two types of GD are described under this group originally as follows:

• Type 2 (OMIM #230900; ORPHA: 77260) is known as acute neuronopathic or infantile GD

 (14, 15), manifests early at infancy (17). It is also the most severe form of GD characterized by CNS involvement (1). Rapid progression with severe deterioration course, leads to death in infancy or early childhood usually before the age of 2 year (17, 20, 21). 

• Type 3 (OMIM #2301000; ORPHA:77261) is known as chronic neuronopathic or juvenile GD or subacute neuronopathic GD (14, 15). It is characterized by less severe CNS involvement (1) and can be confused therefore, with type 1 GD in its early stages (19). It includes several different phenotypes (22) in childhood, adolescence or early adulthood (23-25), and further is divided into subgroups: 3a, 3b, and 3c (26-28). 

***New insights to neuropathic variants***

In fact, because any combination of the GD’s types may occur in any individual patient, it is increasingly recognized that this classification is somewhat non practical (22). Recognition of a subset of patients with type I GD who developed PD (29) and peripheral neuropathies (30), has led to the expansion of phenotype as a clinical phenotype of GD to be considered as a continuum of phenotypes (31). Therefore, it appears that classification of GD needs to be modified periodically as the new variants introduced. Accordingly, we revised current conventional classification to the new expanded classification based on literature (9, 15-17, 27, 30-40) (Figure 1). 

## Epidemiology

Nowadays, GD is classified in “orphan diseases” category, which comprises a group of rare disorders with prevalence of 1:50,000 or lower in the general population (1). The incidence of GD is 1 in 40,000 - 60,000 to 100,000 births in the general population but it could reach up to 1 in 800 - 1,000 in Ashkenazi Jewish populations (5, 41). GD affects men and women equally. According to a report by the National Organization for Rare Disorders, the GD incidence rate may be as high as 1 in 450 births among individuals with Ashkenazi Jewish ancestry and 1:20 000 to 1:200 000 in the general population (42-44). The other forms of GD are uncommon and do not occur more frequently in people of Ashkenazi Jewish descent (45). 

**GD1:** Type 1 is the most common form of the disorder with a prevalence of 1 in 50,000 to 100,000 (19) accounting for 95% of all GD cases (26). It occurs more frequently in people of Ashkenazi (Eastern and central European) Jewish heritage than in those with other backgrounds. This form of the condition affects 1 in 500 to 1,000 people of Ashkenazi Jewish heritage (45). **GD2:** Type 2 GD makes up the minority of GD cases overall. In general, GD has an estimated frequency of 1 in 100,000 to 500,000 live births (46, 47). Like other types of GD, type 2 GD is pan-ethnic in occurrence (33). 

**GD3:** Type 3 GD is also a rare form that affects fewer than 1 in 100 000 people (26-28). 

## Genetic and inheritance pattern

GD is inherited in an autosomal recessive pattern which means both parents must be heterozygote carriers of a mutated gene for production of an affected zygote during conception. 

In each pregnancy, the chance of fetus to have two (i. e., affected) or no (i. e., unaffected) mutated genes is 25%. Moreover, there is a 50% chance with each pregnancy that the offspring have a mutation from one of parents (i. e., heterozygote carrier). Heterozygote carriers typically do not reveal clinical features of the condition (46-49). 

GD is caused by the mutations in the *g*lucocere*b*rosid*a*se (*GBA*) gene on the first chromosome (1q21), composing of 11 exons and 10 introns with 7. 6 kb in length (23). Mutations in the GBA gene cause all three primary forms of GD by altering the stability of the glucosylceramidase or its active site. To date, more than 300 mutations have been described (50). 

## Pathophysiology and Neuropathogenesis

Glycosphingolipids (GSLs) are essential components of eukaryotic cell membranes synthesized in the endoplasmic reticulum and Golgi apparatus and degraded in the lysosomes. They are vital for life (7, 51). 

Glucocerebrosidase also called glucosylceramidase (GlcCerase, GCase) or acid beta-glucosidase is the lysosomal hydrolase coded by the *GBA* gene (3-5). 

Glucocerebroside also known glucosylceramide (GlcCer) is the simplest GSL in the cell membrane of many organs that normally hydrolyzed into glucose and a simpler fat molecule called ceramide by GlcCerase (8, 10). 

**Figure 2:**
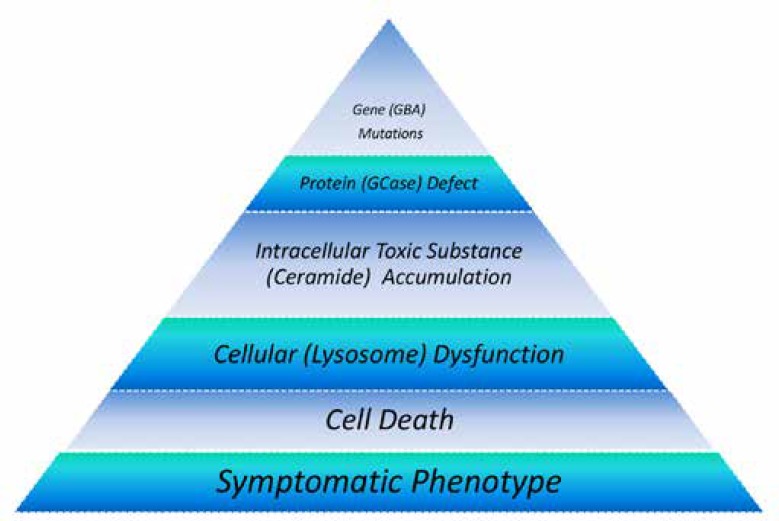
Pathogenesis cascade of GD

Any mutation in the *GBA1* gene causes a diminished activity of GCase. Its deficiency, consequently lead to the accumulation of GlcCer in the lysosomes of macrophages, transforming them into storage cells called Gaucher cells (Figure 2) and cellular dysfunction (52). The exact pathophysiological mechanisms of neurotoxicity are not understood (3) and it is probably different than those of systemic involvement (53). Gaucher cells are found in the brain of patients with neuronopathic GD associated with neuronal loss and glial activation (54, 55). Because the GlcCer turnover in neurons is low, its accumulation is considered significant only when residual GCase activity is notably diminished (56). 

In the neuronopathic GD, Gaucher cells can be found in the perivascular regions and brain parenchyma. In the areas with neuronal loss of the type II GD, brain parenchyma involvement especially in cortical layers III and V, hippocampus brainstem and cerebellum is more frequently reported (54-56). 

## Clinical features

GD may occur at any age, ever fetus and in any humanity as well as geographical districts (16). It is a progressive disorder with a broad spectrum of non-neurological and neurological clinical findings and their variable severity, ranging from neonatal lethal form to asymptomatic subset (16, 20). Variation in the clinical manifestation can be explained by continuum of phenotype (4). All types of GD usually have some degree of visceral involvement with the potential for overlapping manifestations (24). According to the ICGG Gaucher Registry data, the frequency of clinical features for all types of GD include the following as a descending order: Splenomegaly (85%), thrombocytopenia with or without bleeding (68%), hepatomegaly (63%), osteopenia (55%), failure to thrive ( 36%), anemia (34%), bone pain( 33% ), fractures (7%) and bone crises (7%)(16). Pulmonary involvement is rare in all GD phenotypes and seems more frequent in patients homozygous for the 1448G (L444P) mutation (3). 

As mentioned in classification section, conventional and universally-accepted variants of GD include non-neuropathic (type 1 GD) form and neuropathic (type 2 GD and type 3 GD) forms (14-18).

**Figure 3:**
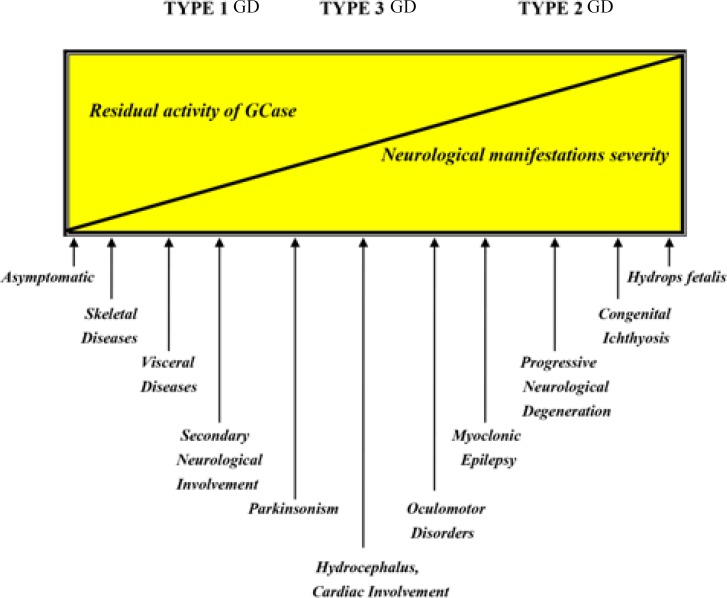
Phenotypic continuum of GD

**I) Non neuropathic GD**

***• Type 1 GD***

Traditionally, lack of nervous system involvement is necessary for a diagnosis of type I GD. However, despite of the customary definition of type 1 GD, some neurological features are reported with this phenotype. Therefore, presence of non-neurological features as exclusively diagnostic criteria of type I GD was questioned (57-67). The recognition of new atypical phenotypes with intermediate features and considering that even patients with type 1 GD can be present with some late-onset neurological presentations, has led to the concept that GD is a continuum of phenotypes (Figure 3) (4-7,39,52). 

1) **Neurological manifestations:** In Type 1 GD, the nervous system may be affected secondarily as a consequence of vertebral involvement resulting from severe hematological and skeletal problems (17, 57). Either vertebral collapse or extraosseous accumulation of Gaucher cells may cause spinal cord compression and/or nerve root compression (17) resulting in secondary neurological complications such as upper motor neuron presentations (57). 

Although there are no primary neurological manifestations in type1GD, certain neurological presentations such as PD and peripheral neuropathy in association with this type have been reported in recent years (3, 17). The frequency of PD (58-60) and symptomatic peripheral neuropathies (61) in type 1GD is higher than in the general population (57). The risk of developing PD in type 1 GD is 5%-7% and 9%-12% before the age 70 and 80 years, respectively (62). The life-time risk of developing PD is an approximately 26-fold higher than the general population (62, 63), often at an earlier age (mean approximately 4–5 yr earlier) (59-61). Moreover, the risk of developing PD may be increased in the heterozygote carrier state. The patients can present with typical features including an asymmetric onset of rigidity, resting tremor, and bradykinesia that is responsive to levodopa (64). It is not evident whether peripheral neuropathy is related to GD, medications or just coincidence (17). Besides, the patients can exhibit atypical and non-motor features such as anxiety, supranuclear oculomotor signs, cognitive impairments, dementia, sleep (REM) disorders, hallucinations and apraxia (58, 64-66). 

2) **Non neurological manifestations:** The clinical presentation is variable; it can be either asymptomatic throughout the life or symptomatic in childhood (2-6). The patients can be present with any symptoms and diagnosed at any age (41). According to the literature, common non neurological clinical features of type 1 GD and their frequency include splenomegaly (90%) (67, 68); thrombocytopenia (60-90%) (69); bone marrow infiltration (80%)(70); hepatomegaly (60-80%)(41); fatigue (50%) (71); anemia (20-50%) (72); growth retardation (34%) (71); gallstones (32%) (73); acute painful bone crises (30%) (72); and avascular necrosis (15%) (74). 

Moreover, there are some uncommon features such as renal involvement, skin involvement, ocular manifestations and vitreo-retinal involvement, myocardial or valvular involvement, insulin resistance and amyloidosis (3). 

**II) Neuropathic GD**

Neuronopathic GD types are rare and constitute about 6% of GD (5% for type 3 GD and 1% for type 2 GD) (35). A definite diagnosis neuronopathic GD (NGD) is best established if there are neurological symptoms and signs in biochemically proven disease while other etiologies have been ruled out (22,75). Neurologic symptoms are characteristic hallmark for types 2 and 3 GD (27). As mentioned in classification section, two types of GD are described under this group. 

## Conventional types

***• Type 2 GD***

Type 2 GD, generally, occurs in infancy with severe systematic presentations specially hepatosplenomegaly and neurological features manifesting with a rapidly progressive course (33). 

***Non perinatal-lethal (Classical or infantile) subtype***

1) **Neurological manifestations:** Neurological features may manifest before splenomegaly or concurrently (52). All patients with type 2 GD experience a rapid neurological deterioration (12). 

The prevalent neurological presentations can be originated from three main anatomical regions including brainstem, pyramidal tracts (corticobulbar and corticospinal tracts) and extrapyramidal tracts (12, 22, 23, 39, 52, 76). Neurodegenerative disease usually appears in six months and progress to a classic picture of disease characterized by triad of “, swallowing and sucking problems, acquired strabismus caused by bilateral 6th nerve palsy and hyperextension of the neck and trunk” (22, 23, 52). The later eventually progresses to opisthotonus, probably due to meningeal irritation (39, 52). Aside strabismus, other oculomotor abnormalities include impaired vision, oculomotor apraxia, Saccadic initiation failure, opticokinetic nystagmus, absence of visual fixation and ophthalmoparesis (76). 

Manifestations of brainstem involvement appear in early infancy before other neurological symptoms and signs (23, 52, 77). The brainstem deterioration proceeds progressively and after a few months the infant ultimately present with either stridor leading to laryngeal obstruction and apnea, or to dysphagia precipitating aspiration (23, 78). Laryngeal stridor is the consequence of bulbar palsy (79-82). 

Adduction and flexion posturing of the thumb, also referred to as “cortical thumb” sign is normal finding in term newborns (79). However, persistent cortical thumb sign beyond 4 months of age considered pathological finding (80). It may be a sign of one of pyramidal tracts involvement, as in type 2 GD (12, 22, 81). Retroflexion of the neck, probably due to laryngomalacia and hypotonia of pharyngeal muscles (12, 22, 52) as well as motor dysfunction including hypertonia, hyperreflexia and expressionless facies or athetosis (23, 33, 39, 52) are other signs of pyramidal tracts involvement. However, children are at first hypotonic and then hypertonic (52, 76). Extrapyramidal rigidity may be seen as well (12, 22). 

Psychomotor development may be delayed initially or regressed after a period of normal development. Some patients, however, continue to acquire new skills despite disease progression 

(12, 52). Other neurological features include universally impaired cognition, progressive microcephaly, arthrogryposis, myoclonic jerks, deafness and epilepsy (12, 39, 76). Seizures occurring later manifest as myoclonic epilepsy that is refractory to antiseizure medications (23, 83, 84). The EEG feature include polyspike discharges seen occipital region predominantly are sensitive to photostimulation; multifocal spike-and-wave paroxysms; and diffuse slowing with high-voltage sharp wave activity during sleep (85). Abnormal brain stem auditory evoked response (BAER) testing, abnormal visual evoked potentials (VEP) and mild cortical atrophy on brain MRI have been reported(76, 83). 

**2) Non neurological manifestations:** Affected newborns often appear normal at birth, but the disease manifests by 3 to 6 months (39, 84). Failure to thrive (30% of cases) may be the first sign makes parents to seek medical attention. It can progress to cachexia in the presence of insufficient nutritional intake (39). Splenomegaly (59% of cases) almost always is the most common finding detected in the onset of disease. Hypersplenism is associated with thrombocytopenia in 60% of cases with or without anemia and leukopenia, usually followed by hepatomegaly (39, 52). Bone involvement is not seen in the type 2 GD, perhaps because there is no sufficient time for the skeletal system involvement (82). Interstitial lung disease occurs due to chronic aspiration, repeated respiratory infections and Gaucher cell infiltration (12, 22, 39, 52). Repeated aspiration and/or prolonged and frequent apnea are the cause of 50% of deaths (23). 

***Perinatal-lethal (Fetal-Neonatal) subtype***

The prevalence of this type is lower than one percent (86). It is the most severe form of GD (37). 

The activity of residual GBA is almost zero (87). The patients can present with either non-immune hydrops fetalis or neonatal ichthyosiform-collodion cutaneous abnormalities (85). 

Perinatal-lethal GD with triad of “hydrops, ichthyosis, and fetal akinesia sequence” has been associated with specific severe mutations (87). 

**1) Neurological manifestations**: Neurological features include signs overlapping with classical type 2 GD including bulbar and pyramidal presentations (12, 22, 23, 39, 52, 76, 81), hypokinesia (43%) with facial dysmorphia (35%) such as low-set ears, a small nose with a flat nasal bridge and anteverted nares) (33), and arthrogryposis plus contractures of distal joints (club foot, camptodactyly) (30%) (23, 88, 89, 90). 

**2) Non neurological manifestations:** Non-neurological feature include prematurity, non-immune hydrops fetalis (22, 88), fetal demise or death usually within the first few days of life (22, 87, 89), hepatosplenomegaly (92%) (23), thrombocytopenia (38%) associated with purpura (22%), anemia (10%), bone marrow involvement with Gaucher cells (88), and neonatal lamellar ichthyosis (collodion baby) phenotype (41%), where infants appear to be covered by a cellophane membrane at birth (12, 88) exhibiting erythematous and shiny skin predominately over the palms and soles or in flexural folds (33). Death occurs secondary to non-neurologicalcomplications including lung hypoplasia caused by plural effusion, hepatic failure, andgastrointestinal tract bleeding (12, 22, 23, 33, 88, 90). 

***• Type 3 GD***

Type 3 GD is more prevalent than type 2 GD (5% versus 1%) due to longer survival in affected patients (52, 91). It is generally associated with later onset in childhood compared with type 2 GD (91), although the patients may present between infancy and adolescence, mostly in the first 5 years of life particularly before the age of two years (52, 82, 92). Therefore, the life span may extend as far as the 5th or even the 6th decade (93, 94). However, they will seek a medical advice during childhood, adolescence, and even adulthood if the non-neurological symptoms are not evident (12, 22), representing variable nature of the patients’ history as well as a phenotypic continuum of type 3 GD (12, 22, 52). 

The clinical expression of type 3 GD can be complex as a combination of visceral presentations of type 1 GD with neurological features of type 2 GD (52, 82). This feature may be challenging as it can lead to difficulty in distinguishing between type 2 GD and type 3 GD, and infrequently even between type 3 GD and type 1 GD (95, 96). It is generally characterized by visceral and less severe CNS involvement (1, 7, 23, 24). The patients may present in childhood with some degree of neurological manifestations as well as hepatosplenomegaly, hematological presentations such as anemia and thrombocytopenia, and osteopathy features such as bone crises and kyphosis (97). Death occurs in patients with severe progressive neurological deterioration, mainly caused by late involvement of the brainstem and related complications such as swallowing problems followed by recurrent aspiration and respiratory compromise (52). 

**1) Neurological manifestations** **:** Type 3 GD shows a slower neurological involvement than type 2 GD (93). The neurological manifestations are a wide spectrum, ranging from mild oculomotor dysfunction to severe and rapidly progressive brain degeneration (52). In the InternationalCollaborative Gaucher Group (ICGG) Registry Study, half of the patients exhibited neurological presentations before two years of old (95). However, in the absence of N370S mutation, neurological features may be evident several years after occurrence of visceral presentations (98). Neurological presentations are a hallmark of type 3 GD (97). 

***1-1)*** ***Neuro-ophthalmic disorders:*** The earliest and most common findings are oculomotor anomalies (about 66% of patients) (52), characterized by a slowing, looping, or failure of the horizontal saccades movements (97, 99). Therefore, ophthalmologists are often primary physicians that visit the patients and then suggest diagnosis of type 3 GD (100). The slowed horizontal saccadic eye movements occasionally are the only neurological symptom (12). Clinically detection of the saccade initiation failure may be difficult. However, it can be simply exposed as absent quick phases by inducing optokinetic and vestibular nystagmus (101). Besides, involvement of vertical saccades may occur (52). 

***1-2) Epileptic disorders:*** The accurate estimation of epilepsy is not known, although it has been reported in 16% of patients in ICGG Registry Study (95). Different seizure types such as generalized and tonic-clonic types have been delineated but myoclonus and progressive myoclonic epilepsy reported more than other cases (95-102). 

Similar to type 2 GD (76, 83, 85), abnormal patterns can be seen in EEG including generalized background slowing or epileptiform discharges as the spike discharges (97, 102). Some patients may present with myoclonic epilepsy without clinically significant visceral storage. Therefore, it can be concluded that GCase deficiency screening should be considered in patients with progressive myoclonic epilepsy in setting of unknown underlying etiology (52). 

***1-3) Cognition and intelligence disorders:*** Cognitive deficits have been reported in 33% of the patients (52). Cognitive impairments typically affect general nonverbal skills with relative sparing of verbal skills (12). As a result, the verbal IQ is typically more than the performance IQ, suggesting a visual-spatial deficits possibly secondary to oculomotor or other motor problems (103). Some patients present with IQ lower than average along with language difficulties, perceptual organization skills, and several learning and functional disabilities (12, 97). Interestingly, patients can occasionally exhibit upper limit normal of IQ scale’s ranges, especially high verbal IQ scores with success in the college and higher degrees (12, 97, 103). 

***1-4) Miscellaneous disorders:*** Behavioral changes, dementia and unexpected death are described in some patients (23). Developmental delay, hearing impairment and other brainstem deficits have been reported. Abnormal “brainstem auditory and somatosensory” evoked potentials have also been noted in some individuals (95). Progressive kyphosis that may develop requiring spinal surgery (23). In the course of disease, progressive cerebellar ataxia or spasticity occur in about 20%–50% of patients affecting walking, and then standing (101). 

**2) Non neurological manifestations:** Similar to type 2 GD, patients with type 3 GD can present with very aggressive visceral disease (104). Therefore, presentation with this phenotype at younger than 2 years of old is frequently associated with type 3 GD (22). Bone involvement is common; severe osteopenia and osteonecrosis of major joints, including the humeral and femoral heads can be a principle cause of morbidity (105). Pulmonary involvement have been reported at least 50% of patients (92). Symptomatic patients usually present with shortness of breath on exercise, coughing, and wheezing. Pulmonary function tests show abnormalities of diffusion, occasionally with a restrictive pattern (22). Other features include hepatomegaly, splenomegaly, anemia and thrombocytopenia, bony pain crisis, bony lytic lesions, bony infarctions, and pathological fractures (106). 

***Subtype 3a GD***

Subtype 3a GD is characterized by mild visceral involvement, but with severe rapidly progressive neurological manifestations including oculomotor apraxia, cerebellar ataxia, spasticity, progressive myoclonic epilepsy refractory to treatment, and dementia (12, 106). 

The prognosis is poor leading to death within the first two decades (13,102). The age of presentation as well as the rate of disease progression are variable (12). 

***Subtype 3b GD***

Unlike the subtype 3a GD, subtype 3b GD is characterized by massive visceral disease and skeletal manifestations but mild, slowly progressive CNS involvement (5, 27, 76, 106). Visceral features include massive hepatosplenomegaly, growth retardation as well as bony symptoms (13, 35, 107). The horizontal supranuclear gaze palsy is the major neurologic sign (27, 35). 

***Subtype 3c GD***

Subtype 3c GD is an atypical rare variant (82) characterized by fatally progressive cardiac valves (tricuspid, mitral or aortic) and ascending aortic calcifications or fibrosis, supranuclear gaze palsy, mild hepatosplenomegaly, corneal opacities and bone disease and hydrocephalus and skeletal anomalies (5, 12, 13, 27, 52). This phenotype was first reported among Arab patients with GD from the Jenin area, but reported among other ethnic groups later (82). It is also exclusively associated with a complete genotype-phenotype correlation across several ethnicities with homozygosity for the D409H (G1342C) mutation (12, 22, 76). Although subtype 3c presents with a unique phenotype, considerable clinical overlap exists between subtypes 3a and 3b (35). They often die in early adulthood (13). 

***Norrbottnian variant***

The subtype 3 Norrbottnian GD is characterized by early onset massive visceral involvement, progressive kyphoscoliosis and mild cognitive deficits (76). Historically, this subtype may have arisen during or before the sixteenth century in northern Sweden (22). This form of GD affects approximately 40% of all known cases in Sweden (12). It is attributed to a founder effect for the L444P mutation (107). The founder effect is the phenomenon in which the high frequency of a specific gene defect within a defined population is explained by common ancestry (i. e., a shared identity by descent) (82). In the classic form of disease, the early clinical characteristics can lead to a diagnosis of type 1 GD (102). The first clinical symptoms occur at the median age of 1 year (12). Visceral involvement characterized by the early onset of prominent hepatosplenomegaly, often requiring splenectomy at an early age (32). Moreover, patients can display hematological symptoms as well as skeletal involvement, often including a gibbous deformity and retinal infiltrates (12). Neurological manifestations include horizontal supranuclear gaze palsy, strabismus due to cranial nerve 6 palsy, ataxia, mild spasticity in the legs, epilepsy as myoclonic or complex partial seizure types, and a slowly progressive cognitive deterioration leading to dementia (108). 

## Non-conventional types

***• Saposin C deficiency***

Saposins are the essential cofactors in multiple stages of the lysosomal degradation of sphingolipids which include a family of four small glycoproteins, known as saposin A, B, C and D (109, 110).These homologous proteins derived from sequential proteolysis of a common precursor protein, prosaposin (PSAP), encoded by the *PSAP* gene on chromosome 10 (111). Saposin C is an established activator for the hydrolytic activity of GCase. It is also plays a protective role in proteolytic degradation of GCase (112). As a result, mutation in saposin c domain of *PSAP* results in saposin c deficiency (111), a very rare cause of GD with normal GCase activity (52).The patients with saposin c deficiency almost always present with clinical presentations similar to those in type 3 GD (108-113).

***•*** ***Type 2-3 intermediate GD***

This non-conventional neuropathic type encompasses patients who survive beyond 2 years of old. They present with intermediary phenotype including clinical features between type 2 and type 3 GD (33). Similar to type 2 GD, the non- neurological presentations are mild to moderate (in contrast to type 3 GD).On the other hand, like to type 3 GD, typical neurological manifestations such as refractory myoclonic epilepsy are frequent which is in contrast to typical neurological features of type 2 GD(33, 52).It seems that several factors such as genetic modifiers, epigenetics, and probably environmental factors play a role not only to intermediate phenotype but also to other atypical GD phenotypes, like type 1 GD association with parkinsonism and peripheral neuropathy discussed earlier (114, 115).

**In Conclusion **unlike previous studies that categorized different types of GD by existence of neurological involvements, based on recent studies, we convinced, there is a continuum range of symptoms in different types and subtypes of GD as type 1 can also be presented by some neurological disorders. Therefore, the neurological symptoms are not the definite criteria for differentiation between different types of gauche disease, and genetic testing is required for confirmation of diagnosis and determining a treatment plan. 
